# Radiation-free CMR diagnostic heart catheterization in children

**DOI:** 10.1186/s12968-017-0374-2

**Published:** 2017-09-06

**Authors:** Kanishka Ratnayaka, Joshua P. Kanter, Anthony Z. Faranesh, Elena K. Grant, Laura J. Olivieri, Russell R. Cross, Ileen F. Cronin, Karin S. Hamann, Adrienne E. Campbell-Washburn, Kendall J. O’Brien, Toby Rogers, Michael S. Hansen, Robert J. Lederman

**Affiliations:** 1 Cardiovascular and Pulmonary Branch, Division of Intramural Research, National Heart Lung and Blood Institute, National Institutes of Health, Building 10, Room 2c713, MSC 1538, Bethesda, MD 20892-1538 USA; 2 Division of Cardiology, Children’s National Medical Center, 111 Michigan Ave, NW, Washington, DC 20010 USA; 3 Division of Cardiology, Rady Children’s Hospital, 3020 Children’s Way, San Diego, CA 92123 USA

**Keywords:** Catheterization, Magnetic Resonance Imaging, Interventional Cardiovascular MRI, Real-time MRI, MRI fluoroscopy

## Abstract

**Background:**

Children with heart disease may require repeated X-Ray cardiac catheterization procedures, are more radiosensitive, and more likely to survive to experience oncologic risks of medical radiation. Cardiovascular magnetic resonance (CMR) is radiation-free and offers information about structure, function, and perfusion but not hemodynamics. We intend to perform complete radiation-free diagnostic right heart catheterization entirely using CMR fluoroscopy guidance in an unselected cohort of pediatric patients; we report the feasibility and safety.

**Methods:**

We performed 50 CMR fluoroscopy guided comprehensive transfemoral right heart catheterizations in 39 pediatric (12.7 ± 4.7 years) subjects referred for clinically indicated cardiac catheterization. CMR guided catheterizations were assessed by completion (success/failure), procedure time, and safety events (catheterization, anesthesia). Pre and post CMR body temperature was recorded. Concurrent invasive hemodynamic and diagnostic CMR data were collected.

**Results:**

During a twenty-two month period (3/2015 – 12/2016), enrolled subjects had the following clinical indications: post-heart transplant 33%, shunt 28%, pulmonary hypertension 18%, cardiomyopathy 15%, valvular heart disease 3%, and other 3%. Radiation-free CMR guided right heart catheterization attempts were all successful using passive catheters. In two subjects with septal defects, right and left heart catheterization were performed. There were no complications. One subject had six such procedures. Most subjects (51%) had undergone multiple (5.5 ± 5) previous X-Ray cardiac catheterizations. Retained thoracic surgical or transcatheter implants (36%) did not preclude successful CMR fluoroscopy heart catheterization. During the procedure, two subjects were receiving vasopressor infusions at baseline because of poor cardiac function, and in ten procedures, multiple hemodynamic conditions were tested.

**Conclusions:**

Comprehensive CMR fluoroscopy guided right heart catheterization was feasible and safe in this small cohort of pediatric subjects. This includes subjects with previous metallic implants, those requiring continuous vasopressor medication infusions, and those requiring pharmacologic provocation. Children requiring multiple, serial X-Ray cardiac catheterizations may benefit most from radiation sparing. This is a step toward wholly CMR guided diagnostic (right and left heart) cardiac catheterization and future CMR guided cardiac intervention.

**Trial registration:**

ClinicalTrials.gov NCT02739087 registered February 17, 2016

**Electronic supplementary material:**

The online version of this article (doi:10.1186/s12968-017-0374-2) contains supplementary material, which is available to authorized users.

## Background

Children with congenital and acquired heart disease often require serial X-Ray cardiac catheterization, accruing significant radiation exposure [1]. Growing and developing children are more radiosensitive than adults, and in children with congenital heart disease undergoing X-Ray cardiac catheterization, radiation-induced chromosomal damage is evident [2–4]. Moreover, children may live long enough to experience oncologic risks of medical radiation [5].

Clinical cardiovascular magnetic resonance (CMR) catheterization was first reported over a decade ago using adjunctive X-Ray [6] and has continued to evolve [7]. We previously described our initial experience with CMR guided right heart catheterization in adults [8]. In systematic comparison of patients undergoing both comprehensive transfemoral right heart catheterization under CMR fluoroscopy and under X-Ray guidance, we found comparable total procedural time and more success entering the left pulmonary artery under CMR. Since that report, our adult clinical center weighed the prospect of direct benefit (radiation-free, additional diagnostic information) and favorable risk profile and classified CMR heart catheterization as the preferred clinical standard for adult patients requiring right heart catheterization.

Similarly, we intend to enable CMR fluoroscopy catheterization as the preferred clinical standard for children. In this report, we pursued complete diagnostic right heart catheterization in children, solely guided by real-time cardiac CMR using commercially available catheters, with the specific aims of feasibility (success/failure, time) and safety (adverse events, heating).

## Methods

### Study Design

#### Research subjects

**Fig. 1:**
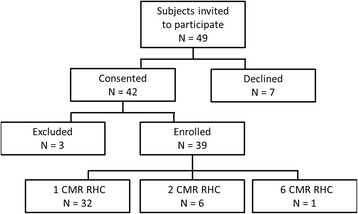
Patient enrollment

The protocol was approved by the Institutional Review Board, and was performed in the combined NHLBI/Children’s National Medical Center MRI catheterization suite at Children’s National Health System in Washington DC (NCT02739087). Patients referred for medically necessary cardiac catheterization were invited to participate in this study (Fig. 1). Patients were excluded for cardiovascular instability, pregnancy, and standard contraindication to CMR scanning (central nervous system aneurysm clip, non-CMR safe or CMR conditional implanted cardiac pacemaker or defibrillator, cochlear implant, etc.). Parental consent (subject assent when appropriate) was obtained for all subjects in writing.

#### Real-time CMR fluoroscopy guided catheterization procedure

**Fig. 2:**
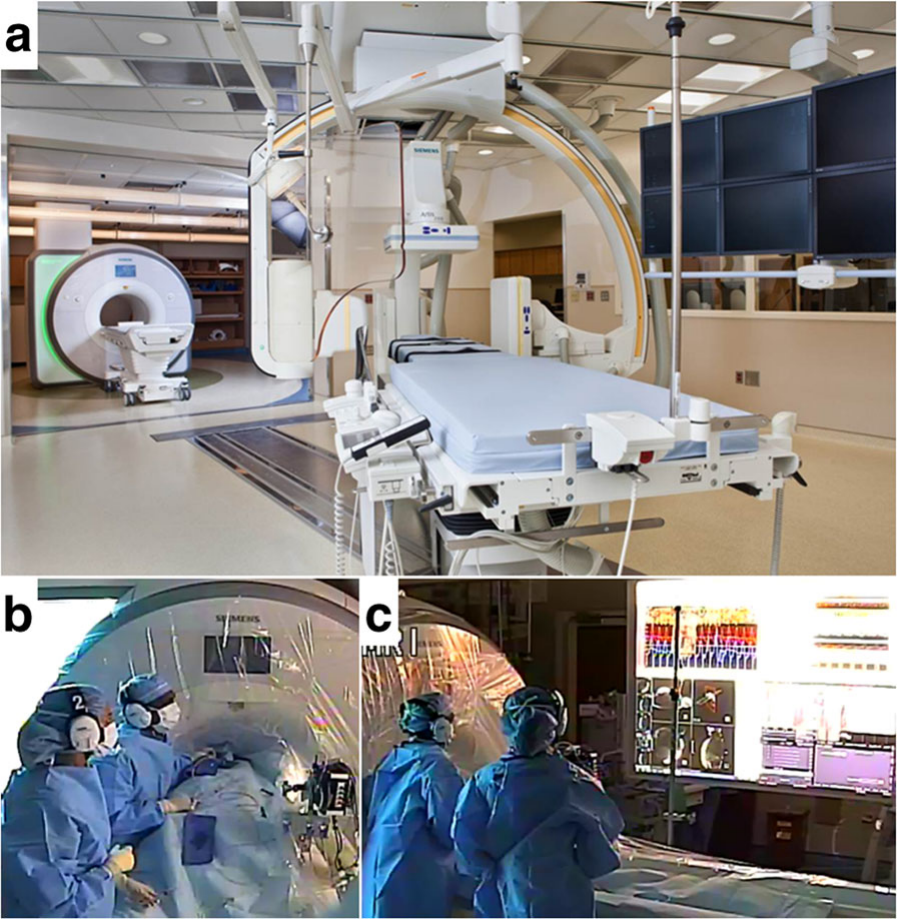
ICMR suite. CMR heart catheterization procedures were performed in an interventional CMR suite (Panel **a**) consisting of adjoining CMR room and biplane X-Ray fluoroscopy suite. The interventional cardiac MRI room is outfitted for invasive cardiac catheterization (Panels **b**, **c**). The patient and CMR scanner have sterile drapes. Operators observe sterile technique and wear noise-cancelling communication headsets. Commercially available passive catheters are connected to conventional pressure transducers that interface (panel **b** *black box*) with the commercial hemodynamic recording system. The room is equipped with commercial projectors that are shielded for CMR operation. Rear projected images show the commercial hemodynamic recording system (panel **c**, *upper left*), and real-time CMR console (panel **c**, *lower left*). Commercial hemodynamic monitor (panel **c**, *upper right*) and CMR host (panel **c**, *lower right*) are also shown


All subjects underwent general anesthesia per institutional clinical standard for cardiac catheterization. General anesthesia (inhaled and/or intravenous) was administered per anesthesiologist discretion as clinically indicated for each patient. Anesthesia and vascular access was obtained in the X-Ray room of the combined interventional cardiovascular magnetic resonance (CMR)/X-Ray fluoroscopy suite (1.5T *Aera and Artis Zee*; *Siemens*, Erlangen, Germany; Fig. 2). Maintaining sterility, the sterile drapes were folded over subjects on a slider for transfer between X-Ray and CMR patient tables. [8] Subjects were then transferred to the CMR scanner for right heart catheterization prior to possible X-Ray guided procedures (left heart evaluation, endomyocardial biopsy, septal defect device closure, etc.) unless procedural workflow dictated otherwise. Heart catheterization was performed under real-time CMR guidance (also known as CMR fluoroscopy, see the Additional file 1: Movie S1) using commercially available balloon-wedge endhole catheters (Additional file 2: Table S1) filled with 1% dilute gadopentetate (*Magnevist* 0.1 mM, Bayer Healthcare, Tarrytown, NY). Right heart catheterization included catheter access to the superior vena cava, inferior vena cava, right atrium, right ventricle, and typically both branch pulmonary arteries including distal pulmonary capillary wedge position. Left heart catheterization, when attempted, was performed by advancing catheter through an atrial septal defect into the left atrium and antegrade across the mitral valve into the left ventricle. Catheters were advanced to ventricular chambers with balloon inflated (without a guidewire) to minimize ventricular ectopy, and without a guidewire absent an CMR-safe commercial option. Continuous simultaneous pressure waveforms confirmed catheter tip localization as is standard practice with traditional X-Ray fluoroscopy.

Additional file 1: Movie S1. Radiation-free CMR guided right heart catheterization. (MP4 3652 kb)


#### CMR room

The CMR room is outfitted for invasive cardiac catheterization as previously described [8] (Fig. 2) with a real-time CMR console (*Interactive Front End*, Siemens, Erlangen, Germany), wireless operator noise-cancelling communication headsets (*IMROC*, Opto-acoustics, Moshav Mazor, Israel), hemodynamic recording system interface (*Physiological Recording in MRI Environment, PRiME*, Centers for Information Technology, NIH, Bethesda, MD), and in-room shielded (GJJ-PRO, Gaven Industries, Saxonburg, PA) LCD projectors to a rear-projection screen that can slide to the head or foot side of the patient as needed.

#### CMR fluoroscopy imaging protocols: catheter guidance

Real-time CMR for catheterization used balanced steady state free precession (SSFP) (TR/TE, 2.7/1.4 ms; flip angle, 45°; bandwidth, 1000 Hz/pixel; matrix 192x144; FOV, 360x360 mm; spatial resolution 1.9 x 1.9 mm; temporal resolution 2.5 – 7.1 frames/second). Parallel imaging (GeneRalized Autocalibrating Partial Parallel Acquisition, GRAPPA) was employed with interactive user selection of acceleration factor (*R*=1-3) in conjunction with the Gadgetron reconstruction engine [9]. Interactive saturation preparation pulses were used to enhance the visibility of gadolinium-filled balloons. The preparation pulse was a flow-sensitive saturation, consisting of 90-180-90 radiofrequency pulses with symmetric gradients around the 180 pulse [10]. The flow sensitive gradients were played out in the slice-select direction, and the field of speed (equal to 2 * venc) was set to 20 cm/sec.

#### CMR imaging protocols: diagnostic imaging

CMR used the following typical parameters. SSFP re-binning [11, 12] for function acquired 20 seconds per slice during free breathing: repetition time (TR)/echo time (TE), 2.8/1.2 ms; flip angle, 50°; bandwidth, 977 Hz/pixel; field of view (FOV), 360x270 mm; matrix, 256x192 pixels; slice thickness/gap, 8/2mm; spatial resolution 1.5 x 1.5 mm; retrospectively gated with 30 cardiac phases.

Velocity-encoded gradient echo: TR/TE 4.8/2.6 ms; flip angle, 20°; bandwidth, 496 Hz/pixel; FOV, 360x270 mm; matrix, 240x135 pixels; slice thickness, 6mm, velocity encoding 200 cm/s; averages, 3; spatial resolution 1.4 x 1.4 mm; acquired with 28.9 ms temporal resolution and interpolated to 30 frames per cardiac cycle for analysis.

### Performance measures and data analysis

Demographics are reported as individual subjects at enrollment. CMR catheterizations are reported as independent events and were evaluated by completion (success/failure), procedure time, and safety events (catheterization, anesthesia). Pre and post CMR body temperature was recorded. Pulmonary and systemic blood flows were measured using both the Fick method and velocity-encoded CMR and indexed for body surface area. The traditional Fick method used subject gender and heart rate to estimate oxygen consumption [13] based on historical data tables. Results are expressed as mean ± standard deviation. A paired student *t-*test compared normally distributed data; two-tailed Wilcoxon signed ranks test compared smaller samples (such as flow in patients with a shunt) (*Excel 2010*, Microsoft, Redmond, WA); *p*<0.05 was considered significant. Flow measurement techniques were compared using Bland-Altman analysis (*Prism* 7.02, Graphpad).

## Results

### Feasibility

#### Procedure success and subject characteristics

In twenty-two months (March 2015 – December 2016), fifty radiation-free transfemoral CMR fluoroscopy guided right heart catheterizations (in 39 subjects; Fig. 1) were performed. All were successful. Forty-nine non-consecutive subjects were invited to participate. Seven declined, and forty-two consented. Three subjects were excluded prior to initiation of CMR heart catheterization: one after CMR localizers identified a metallic object in the abdomen (not detected on standard metal screening history) and two that required alternate vascular access.

One subject developed hemodynamic instability upon induction of general anesthesia, and underwent CMR catheterization on a subsequent day. Seven subjects had multiple CMR guided heart catheterizations (subjects 3, 4, 10, 19, 21, 22 – 2 CMR RHC; subject 8 – 6 CMR RHC) for hemodynamics or suspicion of rejection.

**Fig. 3:**
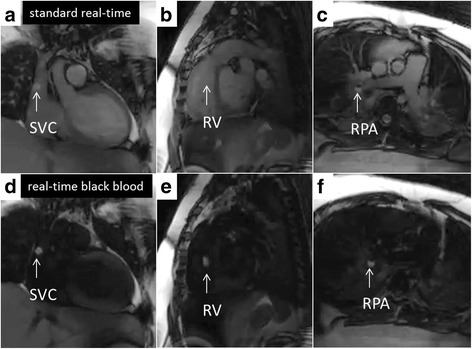
CMR right heart catheterization. Panels (**a**, **b**, and **c**) show real time CMR catheter navigation to superior vena cava (SVC), right ventricle (RV), and right pulmonary artery (RPA) respectively. Panels (**d**, **e**, and **f**) show the same respective imaging planes after flow-sensitive saturation preparation pulse to null blood pool. Gadolinium filled balloon (*white arrow*) is easily and often better (best represented in panel **b** versus panel **e**) visualized during this real-time black blood imaging

Table 1 shows demographic characteristics. The most common indication for catheterization was cardiac transplant surveillance (*n* = 13; 33%). Most (59%) had previous X-Ray cardiac catheterizations and 51% had multiple previous X-Ray cardiac catheterizations (mean 5.5 ± 5 procedures). Many (36%) had known thoracic metallic implants (sternal wires, vascular coils/plugs) including pulmonary artery stents (Fig. 3). Two subjects (5%) were receiving vasopressor infusions for poor myocardial function.

**Table 1:** Demographics

Characteristic	Finding
Age	12.7 ± 4.7
Gender	51% Female
Height	146.3 ± 23.7
Weight	48.8 ± 26.5
Body surface area	1.4 ± 0.5
CMR RHC indication	
Transplant (%)	33
Shunt (%)	28
Pulmonary hypertension (%)	18
Cardiomyopathy (%)	15
Valvular heart disease (%)	3
Other (%)	3
History	
Prior cardiac surgery (%)	44
Prior cardiac catheterization (%)	59
1 previous (%)	8
multiple previous (%)	51
if multiple, how many?	5.5 ± 5
Retained thoracic surgical or catheter device (%)	36
Continuous intravenous vasopressor (%)	5
Oral Medication (%)	72

#### Procedure time and characteristics

Complete radiation-free CMR right heart catheterization was accomplished in all subjects (Table 2). CMR right heart catheterization procedure time was short (12 ± 5 min for one condition; 31 ± 10 min for two conditions). CMR right heart catheterization times in the first half (14 ± 5 min) trended longer than in the second half (11 ± 4 min, *p* = 0.05; one hemodynamic condition) of this experience. All procedures were performed by one of two pediatric interventional cardiologists each with more than five years of experience. One operator had no previous experience with CMR fluoroscopy guidance.

**Table 2:** Procedural characteristics

Procedural detail	Finding
CMR RHC procedure time: 1 condition (min; n = 40)	12 ± 5
CMR RHC procedure time: 2 conditions (min; n = 10)	31 ± 10
Total CMR scanner time (min)	50 ± 14
Additional research CMR imaging (%)	66
Additional clinical CMR imaging (%)	15
CMR RHC body temperature Δ (Celsius)	0.3 ± 0.4
CMR RHC catheters	
Medtronic (%)	50
Edwards "T" tip (%)	40
Arrow (%)	20
Edwards (%)	8
Edwards "S" tip (%)	2
Cook (%)	0
Vascor (%)	0
X-Ray procedure	
X-Ray procedure performed (%)	80
Fluoroscopy time (min)	3.8 ± 3.8
Dose Area Product (Gy∙cm2)	1295.7 ± 2363
Endomyocardial biopsy	23
Left heart catheterization	9
Shunt device closure	10
Coronary angiography	11
Thermodilution	1
Other	3

**Fig. 4:**
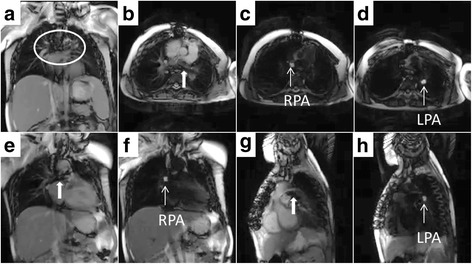
CMR cardiac catheterization in patient with pulmonary artery stent imaging artifact. Panel (**a**) shows imaging artifact (*circled*) from previously placed pulmonary artery stents; CMR right heart catheterization was successful. Panel (**b**) shows oblique axial imaging plane showing branch pulmonary arteries (*thick white arrow* = stent imaging artifact). Panel (**e**) and (**g**) show oblique coronal imaging planes for *right* and *left* pulmonary artery respectively. Panels **c** (RPA = right pulmonary artery), **d** (LPA = left pulmonary artery), **f** (RPA), **h** (LPA) show the same respective imaging planes after flow-sensitive saturation preparation pulse to null blood pool. Gadolinium filled balloon is easily visualized during this real-time black blood imaging

Standard real-time CMR imaging planes were used [8] (Additional files 3, 4 and 5: Figures S1–S3). Five of seven commercially available balloon-wedge endhole catheter types (Table 2; Additional file 2: Table S1) were advanced from femoral venous access. Balloon catheters were inflated with dilute gadolinium contrast to impart CMR visibility in all subjects, none of whom had contraindications to gadolinium exposure in case of balloon rupture. Additional clinically indicated X-Ray procedures were performed in the majority (80%) of cases. Right heart catheterization was not repeated with X-ray guidance. Nine procedures had zero fluoroscopy time. In two subjects [complete atrio-ventricular canal defect (subject 15), atrial septal defect (subject 26)], right and left heart diagnostic catheterization was performed entirely using CMR guidance (Fig. 4). Multiple hemodynamic conditions (i.e., normal saline challenge, 100% inspired oxygen + 40 ppm inhaled nitric oxide) were evaluated in ten subjects. Procedural details are shown in Table 2 *.*

### Safety (adverse events, heating)

Comprehensive radiation-free CMR right heart catheterization was completed on all subjects with no cases of bailout to X-Ray. There were no cases of premature termination of CMR catheterization. There were no safety events related to CMR catheterization or general anesthesia administered in CMR. Subject temperature rise during CMR was minimal (0.3 ± 0.4 degrees Celsius).

### Invasive and imaging data

Combined invasive hemodynamic and imaging findings are summarized in Additional file 2: Table S2. Pulmonary and systemic blood flow (Table 3, Additional file 2: Table S2) were slightly higher (mean difference 0.39 ± 0.67, 95% CI -0.9 to 1.7 L/min/m^2^ and mean difference 0.51 ± 0.66, 95% CI -0.8 to 1.8 L/min/m^2^, respectively) when measured using the Fick technique compared with velocity encoded MRI (Table 3). Table 4 is a list of study subjects.

**Table 3:** Flow measurements using the Fick technique and using velocity encoded CMR

Fick versus Phase Contrast in catheterizations without shunt (*n* = 34)	
Fick pulmonary blood flow (Qp, L/min/m^2^)	3.3 ± 0.7
Fick systemic blood flow (Qs, L/min/m2)	3.3 ± 0.7
Fick pulmonary: systemic blood flow (Qp:Qs)	1 ± 0.1
Fick pulmonary vascular resistance (indexed Woods units)	2.4 ± 1.9
Phase Contrast Main Pulmonary Artery indexed (Qp, L/min/m2)	2.9 ± 0.6*
Phase Contrast Aorta indexed (Qs, L/min/m2)	2.8 ± 0.6*
Phase Contrast pulmonary: systemic blood flow (Qp:Qs)	1 ± 0.1
Phase Contrast pulmonary vascular resistance (indexed Woods units)	2.5 ± 2.1
Fick versus Phase Contrast in catheterizations with shunt (n = 11)	
Fick pulmonary blood flow (Qp, L/min/m^2^)	5.6 ± 2.1
Fick systemic blood flow (Qs, L/min/m2)	3.4 ± 0.8
Fick pulmonary: systemic blood flow (Qp:Qs)	1.8 ± 0.9
Fick pulmonary vascular resistance (indexed Woods units)	1.9 ± 1.6
Phase Contrast Main Pulmonary Artery indexed (Qp, L/min/m2)	5.1 ± 2.1
Phase Contrast Aorta indexed (Qs, L/min/m2)	2.8 ± 0.7*
Phase Contrast pulmonary: systemic blood flow (Qp:Qs)	2 ± 0.9*
Phase Contrast pulmonary vascular resistance (indexed Woods units)	2.1 ± 2

*p<0.05 Student *t*-test (two-tailed)

*p<0.05 Wilcoxon Signed Ranks Test (two-tailed)

**Table 4:** Study subjects

Subject number	Age (yr)	CMR RHC indication	# previous X-Ray Caths	Retained thoracic implant	CMR RHC time (min)
Subject 1	14.8	Valvular heart disease	none	sternal wires	19
Subject 2	21.1	Transplant	15	sternal wires	13
Subject 3	12.8	Transplant	14	sternal wires	19
Subject 4	13.3	Transplant	13	sternal wires	12
Subject 5	17.1	Cardiomyopathy^b^	2	none	48^a^
Subject 6	12.2	Other	none	surgical clips, sternal wires	29
Subject 7	14.9	Shunt (Atrial Septal Defect)	none	none	18
Subject 8	10	Transplant	2	sternal wires	6
Subject 9	5.4	Cardiomyopathy^b^	none	none	12
Subject 10	13.8	Transplant	none	sternal wires	8
Subject 11	9.6	Shunt (Atrial Septal Defect)	none	none	11
Subject 12	10.1	Transplant	2	sternal wires	12
Subject 13	13.1	Pulmonary hypertension	none	none	45^a^
Subject 14	6.3	Transplant	8	surgical clips, sternal wires, pulmonary artery stents, embolization coils	18
Subject 15	10.2	Shunt (Atrioventricular Canal Defect)	2	none	16
Subject 16	18.8	Shunt (Patent Ductus Arteriosus)	none	none	9
Subject 17	15.3	Transplant	2	sternal wires	18
Subject 18	18.2	Cardiomyopathy	none	none	17
Subject 19	15.2	Transplant	2	sternal wires	11
Subject 20	4.4	Shunt (Atrial Septal Defect)	none	none	16
Subject 21	16.6	Transplant	12	sternal wires	15
Subject 22	15.8	Cardiomyopathy	3	none	15
Subject 23	9.2	Cardiomyopathy	1	none	23^a^
Subject 24	17.3	Transplant	13	sternal wires, temporary pacing wire	9
Subject 25	6.3	Shunt (Patent Ductus Arteriosus)	none	none	14
Subject 26	12.5	Shunt (Atrial Septal Defect)	none	none	8
Subject 27	17.5	Transplant	4	surgical clips, sternal wires	13
Subject 28	13.9	Shunt (Patent Ductus Arteriosus)	none	sternal wires	16
Subject 29	5	Transplant	5	none	10
Subject 30	20.7	Shunt (Atrial Septal Defect)	none	none	15
Subject 31	6.1	Pulmonary hypertension	3	none	27^a^
Subject 32	4.6	Pulmonary hypertension	2	none	25^a^
Subject 33	18	Shunt (Atrial Septal Defect)	none	none	9
Subject 34	16	Shunt (Atrial Septal Defect)	none	none	11
Subject 35	9.3	Pulmonary hypertension	5	sternal wires	21^a^
Subject 36	17.3	Pulmonary hypertension	1	none	25^a^
Subject 37	5.7	Cardiomyopathy	4	none	22^a^
Subject 38	14.9	Pulmonary hypertension	0	none	40^a^
Subject 39	13.1	Pulmonary hypertension	5	none	39^a^

Subjects 3, 4, 10, 19, 21, 22: two MRI right heart catheterizations (MRI RHC); Subject 8: six MRI RHC [mean time shown]

^a^Multiple hemodynamic conditions tested in MRI RHC

^b^Continuous vasoactive infusion for poor myocardial function

## Discussion

### Feasibility

We report a series of children undergoing comprehensive radiation-free CMR fluoroscopy guided right heart catheterization, which was successful in all cases with no complications. Two subjects with septal defects had radiation-free CMR right and left heart catheterization. Post-heart transplant was the leading indication for CMR right heart catheterization in our study population. The majority of enrolled subjects had at least one previous X-Ray cardiac catheterization and half of all subjects had multiple previous X-Ray cardiac catheterizations. Many subjects had retained thoracic surgical or catheterization implants. Twenty percent of procedures required testing of multiple hemodynamic conditions; a majority of these procedures were performed with zero fluoroscopy time (70%). A small number of subjects were previously initiated and maintained on continuous vasopressor (milrinone) infusion for poor cardiac function during CMR catheterization. There were no CMR related safety events.

### Toward routine application

This work was a measured step toward routine radiation-free CMR-guided catheterization in children. It is noteworthy that all subjects had successful radiation-free CMR right heart catheterization without complications. Previous work in the field focused on congenital heart disease [6], pulmonary hypertension [14], and adult CMR catheterization [8]. The latter work demonstrated the feasibility of entirely CMR guided right heart catheterization, which is now the clinical standard at our adult institution. We aim to offer the same for children. Diagnostic right and left heart catheterization was demonstrated live at the 2017 Society for Cardiovascular Magnetic Resonance Annual Scientific Sessions (https://www.youtube.com/watch?v=dTXMnEhb7bA) [15].

We developed a clinically realistic workflow, derived from our experience in adults, which included patient transfer, imaging, and catheter manipulation. As a result, procedure times were short. There was a learning curve trend in procedure time comparing early and later procedures [8]. In our lab, inter-modality patient transfer is less than five minutes. CMR blood flow measurements correlated only modestly at rest with blood flow measured using the Fick method and estimated oxygen consumption, as has been reported others [6, 14]. Fick methods lose integrity for flow and resistance measurements during hemodynamic provocations; CMR flow appears unaffected by this limitation [14].

Most hardware and software allowing CMR cardiac catheterization are commercially available except the following non-significant risk devices (used with Institutional Review Board approval): investigational vendor real-time CMR consoles [which are available through a vendor specific research agreement (*Interactive Front End*, Siemens; *iSuite*, Philips) or commercial external controller (Heartvista, Los Altos, CA)] and a high-fidelity hemodynamic recording system interface (self-assembly required; http://nhlbi-mr.github.io/PRiME/) [16]. On-line tutorials are available on how to set up an Interventional CMR suite, videos of cases (including patient transfer), and emerging technology (https://icmr.nhlbi.nih.gov/) [17].

We used many different commercially-available, plastic balloon-wedge endhole catheters for CMR fluoroscopy catheterization. These are the same catheters used worldwide during X-ray fluoroscopy guided catheterization, and were passively-visualized during CMR, meaning CMR conspicuity was imparted by the intrinsic materials characteristics of the devices. These may have limited catheter shaft conspicuity in CMR but are not susceptible to heating (non-metallic). Different commercial offerings have different stiffness and pre-shaped curves. The balloons were filled with dilute Gadolinium contrast in all cases based on our previous adult experience [8] though we were prepared to use air/carbon dioxide for patients with suboptimal glomerular filtration rate or when operator deemed superior buoyancy was required for flow directed catheters. Retained thoracic surgical or catheterization implants with resultant imaging artifact did not preclude successful CMR heart catheterization completion. Patients were excluded for CMR unsafe devices; in the future, we hope select patients may be considered [18]. Continuous vasopressor infusion for poor cardiac function or the need to test multiple hemodynamic conditions did not prevent successful CMR catheterization. The majority (70%) of the latter group were performed without an adjunctive X-Ray procedure (zero fluoroscopy time). All subjects in this series underwent transfemoral access, although we have equipped our CMR catheterization lab with video display capability both at the head and foot side, to allow both transjugular and transfemoral access.

### Interval real-time imaging enhancement

**Fig. 5:**
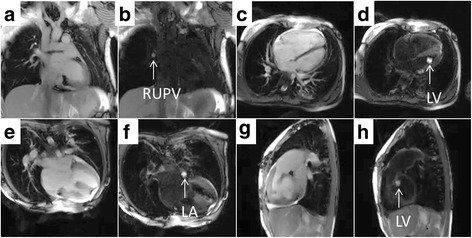
Left heart CMR catheterization in patients with atrial septal defect and complete atrioventricular canal defect. Real-time CMR guided left heart catheterization in patients with atrial septal defect (panels **a**–**d**) and complete atrioventricular canal defect (panels **e**–**h**) are shown. Each two panel sequence (i.e., Panels **a**/**b**, etc.) are in the same imaging plane with standard real-time steady state free precession and after flow-sensitive saturation preparation pulse to null blood pool. Panel (**b**) shows the gadolinium filled balloon navigated from right atrium across the atrial septal defect to the right upper pulmonary vein (RUPV). Panel (**d**) shows the balloon navigated to the left ventricle (LV). Panel (**f**) shows the balloon in the left atrium (LA). Panel (**h**) shows the balloon after navigation to the left ventricle

The flow-sensitive black blood preparation (real-time black blood) imaging sequence improved visualization of the catheter tip (gadolinium-filled balloon) while preserving the surrounding blood and soft tissue imaging (Figs. 3, 4 and 5). In previous work, non-selective saturation preparation was used, which made the balloon conspicuous but also completely suppressed tissue from surrounding structures. Operators would frequently turn the preparation pulse on and off to switch the focus between the balloon and the anatomy. The flow-sensitive black blood preparation enables both the balloon and anatomy to be seen simultaneously, and we found that the operators preferred to use this imaging mode continuously during most of the study. In the future, additional imaging sequences may be helpful as a roadmap to access challenging anatomy or to evaluate catheter based therapeutic procedures.

### Procedural safety

There were no safety events. For quality assurance, we perform quarterly staff drills in role-based emergency resuscitation management, for example, of ventricular arrhythmia or heart block. Evacuation to X-Ray room for potential defibrillation or X-Ray procedure is typically one minute. Nevertheless, no subject required urgent evacuation from the CMR room. Similarly, we simulate emergency patient transfer including: metal screening time out, sterile field maintenance, additional CMR room entry time-out, and anesthesia machine switch. Two anesthesia staff (airway) and two catheterization technologists (patient slider) perform patient transfer. Continuous patient monitoring and high-fidelity hemodynamic recording are displayed simultaneously in X-Ray, CMR, and control rooms. High-fidelity hemodynamic recording system interface filtering minimized magneto-hydrodynamic (MHD) effects on ECG tracing. Additionally, all patients had continuous invasive arterial waveform displayed per institutional protocol. Prolonged real-time CMR may contribute to total body heating, but we observed minimal rise in body temperature during these short CMR catheterization procedures.

### Radiation sparing

The research subjects in this study are suitable for CMR fluoroscopy heart catheterization based on age and pathology. CMR catheterization aims to avoid the hazards of ionizing radiation. While the actual risk of radiation injury remains controversial, even low-level exposure to ionizing radiation is thought to contribute to the long-term risk of malignancy [5, 19] . Growing and developing children are considered more sensitive to radiation and may live longer to experience radiation toxicity. Chromosomal damage is evident in the peripheral blood of children exposed to catheterization-related radiation [2–4]. Children requiring catheterization for congenital heart disease often require multiple lengthy procedures over time. The majority of our cohort required diagnostic or annual surveillance cardiac catheterizations after heart transplant. Five subjects, all post-heart transplant patients, had ten or more previous X-Ray cardiac catheterizations. Six subjects underwent multiple MRI heart catheterizations for suspicion of rejection. These are the very patients that may benefit the most from radiation sparing [1] and concomitant CMR investigation of myocardial (rejection) and perfusion (coronary arteriopathy) abnormalities. Only six subjects had additional clinically indicated cardiac MRI studies in the same setting, but over half had additional research cardiac imaging that referring cardiologists found valuable for management of their patients.

### Limitations

Out of caution, we did not enroll consecutive patients. Instead, we progressively allowed enrollment of younger subjects in three groups: the first ten were limited to age 10 or older; the next ten, age 5 or older (age 5-10, *n* = 3); and the next ten, age 2 or older (age 2-5, *n* = 2; age 5-10, *n* = 5). We found the safety profile of CMR right heart catheterization to be favorable and we intend to be more inclusive. This is a source of potential selection bias. We used widely available commercially available balloon-wedge endhole catheters [8]. They are safe for use in CMR, but can only be visualized at the tip without a guidewire. They are available with variable stiffness, but soften during extended use. These represent potential limitations although in this experience all attempted CMR catheterizations were successful with no safety events.

### Future directions

We plan to offer these procedures systematically to consecutive patients undergoing examination for heart failure, post-heart transplant, cardiomyopathy, and pulmonary hypertension [14] and thereafter to patients before and after Fontan repair [20]. Serial catheterization for patients with single ventricle physiology undergoing staged surgical palliation exposes them to significant radiation [1]. Ultimately, CMR soft tissue visualization is likely to be most beneficial for catheter navigation in structurally abnormal hearts of patients with complex congenital heart disease.

## Conclusions

Radiation-free CMR guided right heart catheterization is feasible and safe in pediatric patients using largely commercially available hardware, software, and catheters. CMR fluoroscopy guidance does not preclude patients with previously implanted metallic implants, continuous hemodynamic vasopressor infusions, or testing needed in multiple conditions. Children requiring multiple, serial X-Ray cardiac catheterizations may benefit most from radiation sparing. This work represents real world application of real-time CMR guidance for routine cardiac catheterization in children. It is an incremental step toward wholly CMR fluoroscopy guided diagnostic cardiac catheterization and in the future CMR guided cardiac intervention.

## Additional files

Additional file 2: Table S1. Balloon-wedge endhole catheter types used. **Table S2.** CMR catheterization data. (DOCX 14 kb)

Additional file 3: Figure S1. CMR Right Heart Catheterization imaging planes: caval view. Real-time CMR acquisition and display console (*Interactive Front End*, Siemens, Erlangen, Germany) shows ideal imaging planes for catheter navigation to inferior and superior vena cava. Imaging planes can be saved as “postage stamps” (left hand column) for “drag and drop” toggling between pre-selected imaging planes. Interactive slice thickness (thick white arrow), saturation preparation (white arrow), and accelerated imaging (asterisk) are important functions for efficient operation. [SVC = superior vena cava; IVC = inferior vena cava]. (TIFF 888 kb)

Additional file 4: Figure S2. CMR Right Heart Catheterization imaging planes: right ventricular outflow tract view. RV = right ventricle; MPA = main pulmonary artery. (TIFF 583 kb)

Additional file 5: Figure S3. CMR Right Heart Catheterization imaging planes: pulmonary artery view. RPA = right pulmonary artery; LPA = left pulmonary artery. (TIFF 571 kb)

## Abbreviations

FOV: Field of view
MRI RHC: Magnetic Resonance Imaging guided Right Heart Catheterization
CMR: Cardiovascular Magnetic Resonance Imaging
SSFP: Steady state free precession
TE: Echo time
TR: Repetition time
