# Supplementary material for: Radiation-free CMR diagnostic heart catheterization in children
Source: J Cardiovasc Magn Reson. 2017 Sep 6;19:65. doi: 10.1186/s12968-017-0374-2 (PMC5585983; doi:10.1186/s12968-017-0374-2)
Supplement: Supplementary file 2 — Balloon-wedge endhole catheter types used. Table S2. CMR catheterization data. (DOCX 14 kb) [file 12968_2017_374_MOESM2_ESM.docx]

**Additional file 1**

Table S1: Balloon-wedge endhole catheter types used

| **Commercial non-metallic MRI catheterization balloon-wedge endhole catheters** |
| --- |
| Teleflex Arrow, Balloon-wedge Pressure Catheter, Model #: AI-07127  5F (60, 110cm), 6F (60, 90, 110 cm), 7F (110 cm), 8F (110 cm) |
| Cook, Flow Directed Balloon Catheter, Model #: FDB5.3-35-80  5.3 F (80 cm) |
| Edwards, True Size Monitoring “S” Tip Catheter, Model #: S111F7  7F 110 cm |
| Edwards, True Size Monitoring Catheter, Model #: 111F7P  7F 110 cm |
| Edwards, True Size Monitoring “T” Tip Catheter, Model #: T111F7  7F 110 cm |
| Medtronic, Pulmonary Wedge Pressure Catheter, Model #: 150075  7F 110cm |
| Vascor, Balloon-wedge Pressure Catheter, Model #: 172-110P  7F 110cm |

Table S2: MRI catheterization data

| **Invasive hemodynamic data [pressures (mmHg), saturation (%)]** | |
| --- | --- |
| Heart rate | 88 ± 19 |
| Arterial mean blood pressure | 68 ± 8 |
| Right atrial mean blood pressure | 9 ± 3 |
| Right ventricular peak systolic pressure | 31 ± 14 |
| Right ventricular diastolic pressure | 10 ± 3 |
| Main pulmonary artery mean blood pressure | 22 ± 10 |
| Right pulmonary artery mean blood pressure | 20 ± 7 |
| Right pulmonary wedge mean blood pressure | 12 ± 4 |
| Left pulmonary artery mean blood pressure | 20 ± 8 |
| Left pulmonary wedge mean blood pressure | 12 ± 4 |
| Arterial hemoglobin saturation | 97 ± 2 |
| Mixed venous hemoglobin saturation | 72 ± 6 |
| Main pulmonary artery hemoglobin saturation | 76 ± 8 |
| Right pulmonary artery hemoglobin saturation | 74 ± 7 |
| Left pulmonary artery hemoglobin saturation | 74 ± 6 |
| Fick pulmonary blood flow (Qp, L/min/m^2^) | 3.8 ± 1.5 |
| Fick systemic blood flow (Qs, L/min/m^2^) | 3.3 ± 0.7 |
| Fick pulmonary: systemic blood flow (Qp:Qs) | 1.2 ± 0.5 |
| Fick pulmonary vascular resistance (indexed Woods units) | 2.3 ± 1.8 |
| **MRI data** | |
| Right Ventricular End Diastolic Volume (ml/m^2^) | 84.3 ± 34 |
| Right Ventricular End Systolic Volume (ml/m^2^) | 41 ± 18.8 |
| Right Ventricular Ejection Fraction (%) | 51.5 ± 8.9 |
| Left Ventricular End Diastolic Volume (ml/m^2^) | 70.9 ± 20.5 |
| Left Ventricular End Systolic Volume (ml/m^2^) | 31.7 ± 13.6 |
| Left Ventricular Ejection Fraction (%) | 56 ± 9.4 |
| Phase Contrast Main Pulmonary Artery indexed (Qp, L/min/m^2^) | 3.4 ± 1.4 |
| Phase Contrast Aorta indexed (Qs, L/min/m^2^) | 2.8 ± 0.6 |
| Phase Contrast pulmonary: systemic blood flow (Qp:Qs) | 1.2 ± 0.6 |
| Phase Contrast pulmonary vascular resistance (indexed Woods units) | 2.4 ± 2.1 |
