# Supplementary figures and images for: Radiation-free CMR diagnostic heart catheterization in children
Source: J Cardiovasc Magn Reson. 2017 Sep 6;19:65. doi: 10.1186/s12968-017-0374-2 (PMC5585983; doi:10.1186/s12968-017-0374-2)

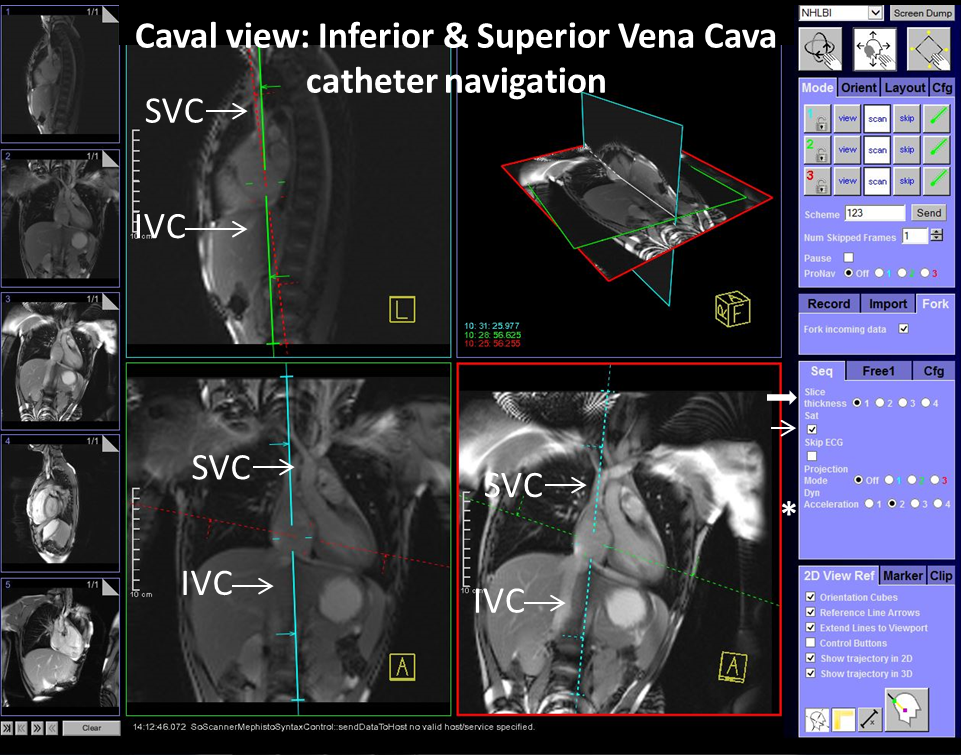

Supplement: Supplementary file 3 — CMR Right Heart Catheterization imaging planes: caval view. Real-time CMR acquisition and display console (Interactive Front End, Siemens, Erlangen, Germany) shows ideal imaging planes for catheter navigation to inferior and superior vena cava. Imaging planes can be saved as “postage stamps” (left hand column) for “drag and drop” toggling between pre-selected imaging planes. Interactive slice thickness (thick white arrow), saturation preparation (white arrow), and accelerated imaging (asterisk) are important functions for efficient operation. [SVC = superior vena cava; IVC = inferior vena cava]. (TIFF 888 kb) [file 12968_2017_374_MOESM3_ESM.tif]

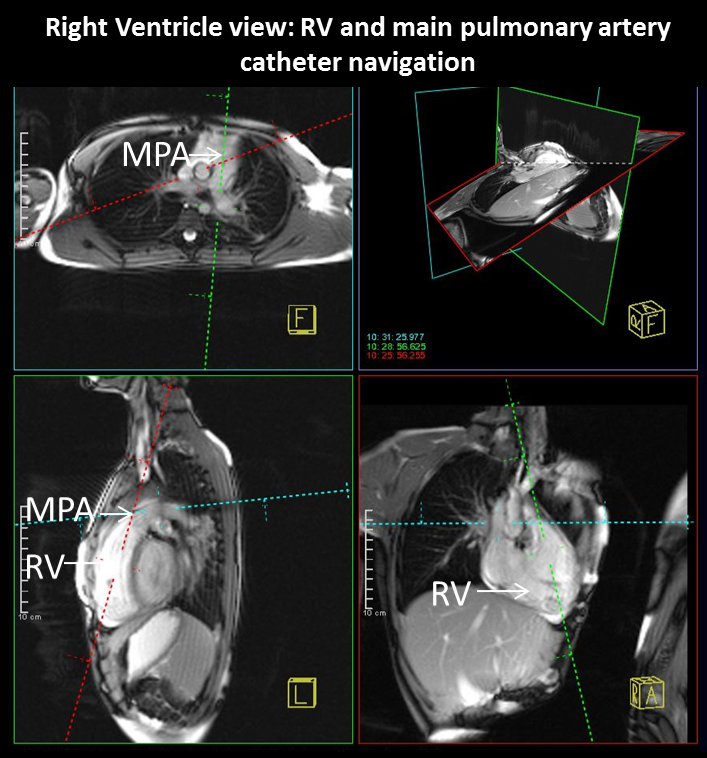

Supplement: Supplementary file 4 — CMR Right Heart Catheterization imaging planes: right ventricular outflow tract view. RV = right ventricle; MPA = main pulmonary artery. (TIFF 583 kb) [file 12968_2017_374_MOESM4_ESM.tif]

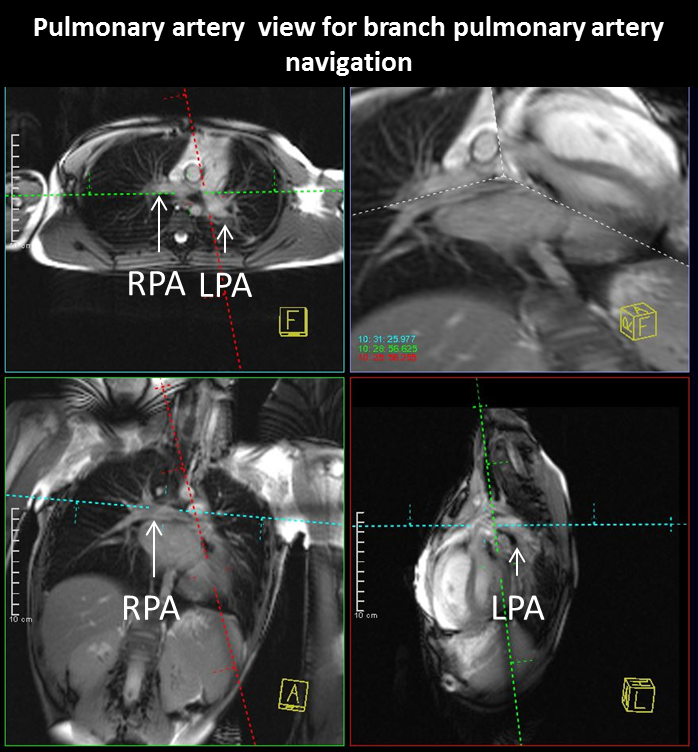

Supplement: Supplementary file 5 — CMR Right Heart Catheterization imaging planes: pulmonary artery view. RPA = right pulmonary artery; LPA = left pulmonary artery. (TIFF 571 kb) [file 12968_2017_374_MOESM5_ESM.tif]
